# Biomedical health profiles of unpaid family carers in an urban population in South Australia

**DOI:** 10.1371/journal.pone.0208434

**Published:** 2019-03-28

**Authors:** Anne F. Stacey, Tiffany K. Gill, Kay Price, Anne W. Taylor

**Affiliations:** 1 Population Research & Outcome Studies, Discipline of Medicine, The University of Adelaide, Adelaide, South Australia, Australia; 2 Adelaide Medical School, The University of Adelaide, Adelaide, South Australia, Australia; 3 School of Nursing and Midwifery, University of South Australia, Adelaide, South Australia, Australia; Iwate Medical University, JAPAN

## Abstract

**Objectives:**

To compare the biomedical health profile and morbidity of adult carers with non-carers.

**Methods:**

The North West Adelaide Health Study (NWAHS) is a representative population-based longitudinal biomedical cohort study of 4056 participants aged 18 years and over at Stage One. Informal (unpaid) carers were identified in Stage 3 of the project (2008–2010). Risk factors, chronic medical conditions and biomedical, health and demographic characteristics using self-report and blood measured variables were assessed. Data were collected through clinic visits, telephone interviews and self-completed questionnaires. Risk factors included blood pressure, cholesterol/lipids, body mass index (BMI), smoking and alcohol intake. Chronic medical conditions included cardiovascular and respiratory diseases, diabetes, and musculoskeletal conditions. Blood measured variables were routine haematology, biochemistry, Vitamin D, and the inflammatory biomarkers high sensitivity C-Reactive Protein (hs-CRP), Tumor Necrosis Factor alpha (TNFα) and Interleukin-6 (Il-6).

**Results:**

The prevalence of carers aged 40 years and over was 10.7%, n = 191. Carers aged 40 years and over were more likely to assess their health status as fair/poor and report having diabetes, arthritis, anxiety and depression. They also reported insufficient exercise and were found to have higher BMI compared with non-carers. Significant findings from blood measured variables were lower serum Vitamin D and haemoglobin. Male carers had raised diastolic blood pressure, higher blood glucose, lower haemoglobin and albumin levels and slightly elevated levels of the inflammatory biomarkers TNFα and hs-CRP.

**Discussion and conclusions:**

This study confirms informal carers had different biomedical profiles to non-carers that included some chronic physical illnesses. It identifies that both female and male carers showed a number of risk factors which need to be considered in future caregiver research, clinical guidelines and policy development regarding carer morbidity.

## Introduction

Although research findings in the caregiving literature have been mixed and at times contradictory, providing long-term care of persons with disability, physical, mental health illnesses and frailty, has been associated with higher rates of hypertension, heart disease, arthritis and other chronic conditions in informal family carers [1–7]. The psychological impact of informal caregiving on carer health, which has received greater emphasis than chronic conditions in the caregiving literature, has indicated that carers frequently experience stress, distress, anxiety and depression, particularly female carers who usually represent the majority of carers [2,8–13]. Published research has mostly been based on self-reported data, while biomedical profiles of carer health that include clinic measured physical and physiological data have been slower to emerge, especially those using well-designed population studies.

### Population studies based on self-report data

National surveys of family carers from the United Kingdom, Europe, Canada, Australia and other countries have described some international trends of carer morbidity at the population level [14–19]. They have found independent associations of chronic exposure to informal caregiving and self-reported poor health even at the extremes of the age range, in both younger and older carers [20]. In recent years surveys of the public health impacts of caregiving in the United States of America (USA) indicate family carers have had a slight to modest decline in their health [21,22]. Carers also reported chronic medical conditions such as diabetes, and joint pain was identified as a recurrent health problem. Assessing risk factors among carers revealed responsible health behaviours in relation to taking exercise and checking cholesterol levels, and those carers surveyed were less likely to be current smokers [23].

### Biomedical measures of carer health: Inflammatory biomarkers

Biomedical studies in the caregiver literature examining inflammatory, immunological and metabolic profiles of carers include mainly small clinical studies. Some of these demonstrated associations between informal caregiving and altered biomarkers in carers of persons with stroke, cancer or the frail aged [4,24]. More detailed physiological assessments of carers’ health have revealed elevated levels of pro-inflammatory biomarkers, in particular plasma cytokines such as Interleukin (IL-6), high sensitivity C-Reactive Protein (hs-CRP) and Tumor Necrosis Factor alpha (TNFα) [25–27]. There have been mixed results from other studies of biomarkers among carer participants. For example a recent review of the literature (2017) found only weak associations between caregiving, stress and increased pro-inflammatory biomarkers, such as IL-6 and CRP among spousal and female home based relatives caring for older persons. The carers themselves were often aged sixty years and over [28]. Another systematic review that was specific to the psychobiological impact of dementia caregiving had a focus on chronic stress and incorporated a broad range of biological markers [29]. An overview of risk factors in carers confirmed differences in blood pressure and heart rate between carers and non-carers, also Body Mass Index (BMI) and weight gain were reported to be different between male and female carers [30]. Caregiving stress was found to be moderated by gender [30] while an earlier study had reported that the negative impact of caregiving on health was not observed in individuals who did not find caregiving to be stressful. [7]. As much past research has been based on dementia caregiving and stress in carers, there is a lack of population research which can provide a broader profile of carer health characteristics and offer a different perspective of the distribution of chronic disease among informal carers.

This study therefore aimed to compare general and biomedical health status of informal carers with non-carers from the same population with an emphasis on gender differences. Access to comprehensive self-reported and biomedical data from the North West Adelaide Health Study (NWAHS) made our investigation possible and provided a wider selection of haematological and biochemical blood variables rarely featured in carer projects. Research objectives were to analyse a range of risk factors and selected chronic medical conditions, using both self-report and clinically measured blood and other biomedical variables, including a selection of inflammatory biomarkers. The research questions were: *Do informal family carers show different biomedical profiles in terms of blood and other measured variables than non-carers*? and secondly; *Is there an association between the caregiving role*, *risk factors and chronic conditions amongst South Australian informal carers*?

## Methods

### Study design and setting

The NWAHS is a representative population based longitudinal cohort study set in the north and western suburbs of Adelaide, which is the capital of South Australia. The northern and western regions of Adelaide number approximately half of the city’s population and one quarter of South Australia’s population. These regions reflected the demographic profile of the state’s population at the time of the initial data collection. The study was designed to investigate the prevalence of a number of chronic conditions and health-related risk factors along a continuum, from not at risk, to at risk, to diagnosed, to co-morbidity to death. Stage 1 occurred between 1999 and 2003, Stage 2: 2004–6 and Stage 3: 2008–10. The full methodology of the NWAHS, including original sample selection procedure, entry and exclusion criteria, original interview schedules and biomedical measurements have been comprehensively described and published elsewhere [31,32].

### Study population and participants

Initially 4,056 participants aged 18 years were randomly selected and recruited by telephone interview in Stage 1. The analysis for this paper focuses on data collected from Stage 3 only (2008–2010). Stage 3 was the most recent relevant data collected which included both bio-medical data and carer status. Data collection at Stage 3 included (1) a Computer Assisted Telephone Interview (CATI); (2) a self-completed questionnaire; (3) a biomedical examination at a clinic. Overall 2,487 (67%) of the eligible sample completed all of these assessments. The main focus of our study compared health risk factors, chronic medical conditions and biomedical health characteristics with non-carers, using self-report, clinic and blood measured variables. Those aged over 40 years were included in this study as testing for all of the inflammatory biomarkers was only conducted on this group.

### Self-reported variables

In order to determine the prevalence of carers within the cohort, participants were asked as part of the self-complete questionnaire:

*Do you provide long-term care at home for a parent*, *partner*, *child*, *other relative or friend*, *who has a disability*, *is frail*, *aged or who has a chronic mental or physical illness*?

Demographic characteristics selected for this study included age, sex, marital status, work status, educational status, annual household income, and employment status (including whether participants received government support from age, carer or disability pensions). Participants self-reported if they had ever been diagnosed by a doctor for arthritis, cardiovascular (CVD) (ie heart attack, stroke, angina, transient ischaemic attack), or a mental health condition (i.e. anxiety, depression, stress related problem).

Smoking was assessed using standard questions which related to current smoking and the frequency of smoking and alcohol consumption was determined from questions based on the National Heart Foundation Risk Factor Prevalence Study undertaken in 1989 [33]. Physical activity was determined from the amount of walking, moderate and/or vigorous activity undertaken over a one week period, which was then categorized into “No activity”, “Insufficient activity” (less than 150 minutes of walking, moderate and/or vigorous activity) and “Sufficient” (150 minutes or more per week) [34]. General health was assessed using the SF1, which is the first question of the Short Form 36 (SF36) [35].

### Clinic measured variables

The presence of diabetes was derived from a self-reported doctor diagnosis of diabetes and/or a fasting plasma glucose level of greater than or equal to 7.0 mmol/L. The presence of asthma was determined using self-reported, doctor diagnosed asthma and spirometry measures following administration of salbutamol. For example a change in Forced Expiratory Volume in one second, (FEV1) > = 12% & >200ml, or absolute change greater or equal to 400ml from baseline measurements [36,37].

Other clinically measured risk factors included blood pressure readings, height and weight for calculation of BMI, and waist and hip circumference measurements to determine waist/hip ratio (WHR) using standardized measurement techniques. Details of procedures for measuring and techniques have been described and published elsewhere [38–40]. BMI was categorized according to the World Health Organization (WHO) criteria and a high WHR was defined as > 1.0 for males and >0.85 for females [41,42].

A fasting blood sample was collected by venipuncture from all participants who were able to provide an adequate amount of blood sample at the clinic visit. Samples were transported to an accredited National Association of Testing Associations (NATA) laboratory for analysis. Biochemical measurements of hs-CRP, glucose and albumin levels were determined using an Olympus AU5400 (Beckman Coulter, USA) and glycosylated haemoglobin (HbA1c) using a Bio-Rad Variant II (HPLC) (Bio-Rad, USA). High density lipoprotein (HDL) and total cholesterol were analysed using an Olympus AU5402. Both low density lipoprotein (LDL) and the total cholesterol/HDL ratio were calculated from these results. Haemoglobin (Hb) levels were determined using a Sysmex XE (Japan). Vitamin D levels to April 2010 were determined using and enzyme Immunoassay method from Immunodiagnostic Systems (IDS, UK) and performed on a BEST 2000 automated enzyme-linked immunosorbent assay (ELISA) system from Biokit. From April 2010, Vitamin D was measured using and automated Chemiluminescent assay from IDS and performed on an iSYS Automated Immunoassay system from IDS. The patient comparison during the change over gave a Passing-bablock regression equation of y = -1.61 + 1.07x with a bias of -1.9nmol/L indicating good agreement.

The fasting blood sample measured a series of inflammatory biomarkers in study participants aged 40 years and over. IL-6, TNFα, e-Selectin (e-Sel) and Myeloperoxidase (MPO) levels were measured with an ELISA and Cobas autoanalyzer (Roche Diagnostics US).

### Data weighting

In Stage 1, data were weighted by region (western and northern health regions), age group, sex and probability of selection in the household to the Australian Bureau of Statistics 1999 Estimated Resident Population and the 2001 Census data [43,44]. Weighting was undertaken to reflect the population of interest and to correct for potential non-response bias in which some demographic groups of respondents may be over- or under-represented. Stage 3 was reweighted using the 2009 Estimated Resident Population for South Australia and incorporated participation in the three components (CATI), self-complete questionnaire, clinic), whilst retaining the original weight from Stage 1 in the calculation. All analyses in this paper, where applicable, are weighted to the population of the northern and western suburbs of Adelaide.

### Data analysis

Statistical analysis was conducted using SPSS version 24 (IBM, Armonk, NY, USA) and STATA version 14 (StataCorp, College Station, TX, USA). Descriptive analysis (proportions, means, medians where applicable) were determined for all of the predictor variables (demographic characteristics, chronic conditions and health risk factors). Bivariable analysis using chi-square tests and including post hoc tests using the adjusted residuals, were used to determine which categories were significantly different from the other categories, combined for both carers and non-carers. All continuous data were tested for normality using Kolmogorov-Smirnov and Shapiro-Wilk tests (both tests were used to obtain a more in depth understanding of whether data were normally distributed), and data that were not normally distributed were analysed using non-parametric tests (Mann-Whitney U).

Generalised linear models using the binary outcome variable of presence carer or not a carer were used with the “svy” estimators in STATA and weighted data to determine the relative risks (RR) of each of the predictors, in association with the outcome variable. Separate multivariable models were created for males and females which included all possible predictors.

### Ethical approval

All protocols and procedures were approved by the Human Research Ethics Committee of The Queen Elizabeth Hospital, in Adelaide, South Australia, and all participants provided written informed consent.

## Results

The prevalence of carers aged 40 and over was 10.7% (95% CI 9.3–12.3), n = 191. Table 1 presents the demographic characteristics for carers aged 40 years and over compared to non-carers. Carers were more likely to be female, married and have a lower education level. They were also more likely to be retired, undertake home duties or were unable to work. Carers had higher levels of uptake of carer pensions, age pension and disability pension. Carers were also more likely to be over 60 years of age and have an annual income of between $20,000 and $40,000 per year.

**Table 1:** Demographic characteristics of carers compared to non-carers, aged 40 years and older.

Variable	Carers			Non-carers			p value
	n	%	95% CI	n	%	95% CI	Χ^2^
Gender							
Male	78	9.1 ↓	7.3–11.3	779	90.9 ↑	88.7–92.7	
Female	113	12.1 ↑	10.1–14.5	818	87.9 ↓	85.5–89.9	0.038
Age Group (years)							
40–59	92	9.1 ↓	7.3–11.3	921	90.9 ↑	88.7–92.7	
60 years and over	99	12.8 ↑	10.7–15.2	676	87.2 ↓	84.8–89.3	0.011
Marital status							
Married/de facto	150	11.8 ↑	10.1–13.8	1122	88.2 ↓	86.2–89.9	
Divorced/Separated	9	5.3 ↓	3.2–8.7	160	94.7 ↑	91.3–96.8	
Widowed	10	6.1	2.7–13.2	146	93.9	86.8–97.3	
Never Married	11	12.6	6.8–22.2	73	87.4	77.8–93.2	0.015
Employment status							
Self/ Full time / Part time	63	6.6 ↓	5.1–8.6	883	93.4 ↑	91.4–94.9	
Unemployed	5	19.4	7.9–40.2	21	80.6	59.8–92.1	
Home duties	13	19.3 ↑	11.3–31.0	53	80.7 ↓	69.0–88.7	
Retired	74	13.3 ↑	10.8–16.3	482	86.7 ↓	83.7–89.2	
Student/Volunteer	2	13.0	3.4–38.9	12	87.0	61.2–96.6	
Unable to work	14	21.6 ↑	12.6–34.5	52	78.4 ↓	65.5–87.4	
Carer	8	100.0 ↑	-	-	-	-	<0.001
Educational status							
High school	122	13.3 ↑	11.2–15.8	795	86.7 ↓	84.2–88.8	
Trade/ Certificate/ Diploma	42	8.0 ↓	5.9–10.7	482	92.0 ↑	89.3–94.1	
Bachelor degree or higher	15	6.3 ↓	3.7–10.4	226	93.7 ↑	89.6–96.3	<0.001
Annual household income ($Aus)							
Up to $20,000	22	10.8	7.2–15.8	180	89.2	84.2–92.8	
$20,000-$40,000	75	18.3 ↑	14.9–22.3	332	81.7 ↓	77.7–85.1	
$40,000-$60,000	16	5.9 ↓	3.8–9.1	259	94.1 ↑	90.9–96.2	
$60,000-$80,000	20	9.4	5.8–14.8	193	90.6	85.2–94.2	
$80,000-$100,000	15	8.5	4.7–15.0	157	91.5	85.0–95.3	
More than $100,000	12	4.2 ↓	2.3–7.6	272	95.8 ↑	92.4–97.7	
Not stated	20	15.3	9.7–23.3	112	84.7	76.8–90.3	<0.001
Carer Payment							
No	63	6.2 ↓	4.7–8.0	959	93.8 ↑	92.0–95.3	
Yes	26	86.7 ↑	64.7–95.9	4	13.3 ↓	4.1–35.3	<0.001
Age Pension							
No	63	6.2 ↓	4.7–8.0	959	93.8 ↑	92.0–95.3	
Yes	64	15.1 ↑	12.1–18.8	357	84.9 ↓	81.2–87.9	<0.001
Disability Pension							
No	63	6.2 ↓	4.7–8.0	959	93.8 ↑	92.0–95.3	
Yes	14	16.8 ↑	10.1–26.5	69	83.2 ↓	73.5–89.9	<0.001

Chi square post hoc tests ↑↓ indicates statistically significantly difference in categories using adjusted standardised residual

Table 2 presents bivariable analysis of general health, risk factor and chronic conditions of carers aged 40 years and over, compared to non-carers. Carers were more likely to have higher BMI and WHR than non-carers, were less likely to undertake a sufficient level of physical activity but had a lower alcohol risk. Carers were also more likely to have diabetes, arthritis, anxiety, depression and fair/poor health status compared to non-carers.

**Table 2:** Risk factor and chronic condition profile of carers compared with non-carers, aged 40 years and over.

Variable	Carers			Non-carers			p-value
	n	%	95% CI	n	%	95% CI	Χ^2^
Body Mass Index							
Underweight/ normal	29	7.1 ↓	5.0–10.0	381	92.9 ↑	90.0–95.0	
Overweight	76	11.1	8.9–13.9	602	88.9	86.1–91.1	
Obese	72	12.2	9.8–15.2	515	87.8	84.8–90.2	0.027
Waist-to-hip ratio							
Normal	102	9.1 ↓	7.5–11.0	1025	90.9 ↑	89.0–92.5	
High	76	13.6 ↑	11.0–16.8	481	86.4 ↓	83.2–89.0	0.004
Smoking status							
Non smoker	91	11.7	9.0–13.6	733	88.9	86.4–91.0	
Ex-smoker	73	10.4	8.4–12.8	625	89.6	87.2–91.6	
Current smoker	27	10.7	7.3–15.5	224	89.3	84.5–92.7	0.919
Alcohol Risk							
Non drinker, no risk	112	12.2	10.2–14.6	807	87.8	85.4–89.9	
Low risk	60	9.9	7.7–12.6	551	90.1	87.4–92.3	
Intermediate to very high	3	3.6 ↓	1.2–9.8	72	96.4 ↑	90.2–98.8	0.041
Physical Activity							
No activity	49	14.1 ↑	10.5–18.7	295	85.9 ↓	81.3–89.5	
Activity but not sufficient	71	12.5	10.0–15.6	498	87.5	84.4–90.0	
Sufficient activity	58	7.6 ↓	5.9–9.7	704	92.4 ↑	90.3–94.1	0.001
Asthma							
No	134	9.9	88.3–91.6	1218	90.1	88.3–91.6	
Yes	44	13.2	82.5–90.1	288	86.8	82.5–90.1	0.079
Cardiovascular disease							
No	158	10.3	8.9–12.0	1370	89.7	88.0–91.1	
Yes	22	13.8	8.7–21.1	135	86.2	79.0–91.3	0.185
Diabetes							
No	149	10.0 ↓	8.5–11.6	1344	90.0 ↑	88.4–81.5	
Yes	29	15.3 ↑	10.8–21.3	162	84.7 ↓	78.7–89.2	0.022
Arthritis							
No	95	9.1 ↓	7.4–11.2	945	90.9 ↑	88.8–92.6	
Yes	75	13.7 ↑	11.1–16.8	471	86.3 ↓	83.2–88.9	0.005
Anxiety							
No	140	9.9 ↓	8.4–11.7	1271	90.1 ↑	88.3–91.6	
Yes	18	19.2 ↑	12.0–29.2	76	80.8 ↓	70.8–88.0	0.005
Depression							
No	133	9.8 ↓	8.3–11.6	1222	90.2 ↑	88.4–91.7	
Yes	25	16.5 ↑	10.8–24.5	125	83.5 ↓	75.5–89.2	0.011
Stress							
No	145	10.2	8.6–11.9	1288	89.8	88.1–91.4	
Yes	12	17.1	10.3–27.1	58	82.9	72.9–89.7	0.062
SF1							
Ex/very good/good	133	9.3 ↓	7.9–11.0	1287	90.7 ↑	89.0–92.1	
Fair/poor	57	16.2 ↑	12.5–20.7	293	83.8 ↓	79.3–87.5	<0.001

Chi square post hoc tests ↑↓ indicates statistically significantly difference in categories using adjusted standardised residual

Table 3 presents a comparison between carers and non-carers for clinic measured variables (blood pressure and blood measured tests). Significant differences were evident between carers and non-carers for the blood measured variables hs-CRP, HbA1c, Hb, and Vitamin D (Table 3). There were no significant differences with regard to the other inflammatory biomarkers IL-6, MPO, TNFα, and e-Sel.

**Table 3:** Clinic measured variables, carers compared with non-carers, aged 40 years and over.

	Carer				Non-carers				
	n	Mean	SD	Median	n	Mean	SD	Median	p-value
Systolic BP	178	129.5	16.8	128.0	1505	129.4	19.2	127.0	0.568
Diastolic BP	178	78.0	8.6	78.0	1505	77.7	8.7	77.5	0.594
CRP	173	4.6	7.8	2.3	1490	3.4	4.7	2.0	0.015
HbA1c	176	6.0	0.8	5.8	1490	5.8	0.8	5.7	0.007
LDL	176	3.1	1.0	3.1	1473	3.1	1.0	3.0	0.405
HDL	176	1.5	0.4	1.4	1492	1.5	0.4	1.4	0.191
Total cholesterol	176	5.2	1.1	5.2	1492	5.3	1.1	5.2	0.755
Total cholesterol/HDL ratio	176	3.7	0.9	3.6	1492	3.7	1.1	3.6	0.076
Glucose	176	5.4	1.2	5.1	1490	5.3	1.2	5.1	0.125
Hb	175	139.4	14.3	139.9	1489	142.8	13.3	143.0	0.014
Vitamin D	176	64.7	25.5	62.0	1466	70.1	27.9	66.0	0.009
Albumin	175	39.4	3.2	39.5	1491	39.8	3.2	40.0	0.111
Il6	152	1.9	1.8	1.4	1220	1.7	1.6	1.2	0.352
MPO	152	218.6	229.4	143.4	1219	202.4	237.2	118.8	0.172
TNFα	152	2.2	3.8	1.6	1220	1.8	2.6	1.4	0.106
E-selectin	152	32.8	11.8	31.9	1219	32.9	16.7	30.2	0.796

Non-parametric tests undertaken for non-normally distributed data

Table 4 presents the results of the multivariable models for males and females. Pension type (carer, aged, disability) was excluded from the analysis, as were total cholesterol and total cholesterol/HDL ratio due to collinearity. Male carers compared with non-carer males were more likely not to be employed (RR 2.52, 95% CI 1.19–5.31; p = 0.015); undertake some activity (RR 2.21, 95% CI 1.22–4.00; p = 0.009); have lower systolic (RR 0.96, 95% CI 0.94–0.99; p = 0.011) but higher diastolic blood pressure (RR 1.13, 95% CI 1.07–1.20; p = <0.001). Male carers were also more likely to have higher levels of blood glucose (RR 1.40, 95% CI 1.03–1.89; p = 0.03), raised hs-CRP (RR 1.03, 95% CI 1.00–1.06; p = 0.023) and TNFα (RR 1.12, 95% CI 1.06–1.20; p = <0.001) but lower levels of HbA1c (RR 0.54, 95% CI 0.33–0.89; p = 0.016) and albumin (RR 0.90, 95% CI 0.82–1.00; p = 0.040). Female carers were less likely to be widowed, separated or divorced and to have lower levels of income below $40,000 when compared with non-carer females.

**Table 4:** Relative risk of predictor variables associated with being a carer compared to non-carers, male and female aged 40 and over.

	Male		Female	
	RR (95% CI)	*p*-value	RR (95% CI)	*p*-value
Marital status				
Never married	1.00		1.00	
Widowed	1.10 (0.08–15.39)	0.941	0.23 (0.07–0.77)	0.017
Separated/divorced	1.62 (0.16–16.27)	0.684	0.19 (0.06–0.64)	0.007
Married/ de facto	2.88 (0.32–26.13)	0.347	0.85 (0.36–2.01)	0.714
Annual household income				
More than $100,000	1.00		1.00	
$80,000-$100,000	1.16 (0.27–4.92)	0.842	3.09 (0.52–18.51)	0.217
$60,000-$80,000	1.05 (0.28–3.93)	0.938	3.80 (0.66–21.77)	0.134
$40,000-$60,000	0.59 (0.17–2.08)	0.411	1.56 (0.25–9.88)	0.638
$20,000-$40,000	1.49 (0.49–4.53)	0.478	6.64 (1.29–33.18)	0.024
Up to $20,000	3.24 (0.74–14.25)	0.119	7.59 (1.29–44.76)	0.025
Not stated	0.96 (0.16–5.65)	0.966	5.65 (1.07–29.78)	0.041
Employment status				
Self/ Full time employed/Part time employed	1.00		1.00	
Not employed	2.52 (1.19–5.31)	0.015	1.55 (0.84–2.86)	0.159
Educational status				
Bachelor degree or Higher	1.00		1.00	
Trade/ Certificate/ Diploma	0.92 (0.37–2.33)	0.862	1.18 (0.36–3.90)	0.780
High school	1.17 (0.42–3.23)	0.763	1.58 (0.52–4.85)	0.420
Body Mass Index				
Underweight/normal	1.00		1.00	
Overweight	1.20 (0.41–3.53)	0.734	1.51 (0.74–3.05)	0.780
Obese	1.54 (0.53–4.48)	0.427	1.18 (0.54–2.60)	0.420
Waist:Hip ratio				
Normal	1.00		1.00	
High	0.65 (0.29–1.47)	0.304	1.23 (0.77–1.97)	0.379
Smoking status				
Non smoker	1.00		1.00	
Ex smoker	0.90 (0.47–1.72)	0.747	1.11 (0.71–1.74)	0.650
Current smoker	1.46 (0.46–4.69)	0.522	1.14 (0.51–2.56)	0.746
Alcohol Consumption Risk				
High risk	1.00		1.00	
Low risk	7.01 (0.85–57.47)	0.070	2.17 (0.57–8.17)	0.254
Non drinkers / no risk	6.06 (0.75–48.62)	0.090	3.29 (0.86–12.59)	0.082
Recreational physical activity				
Sufficient	1.00		1.00	
Activity but not sufficient	2.21 (1.22–4.00)	0.009	1.04 (0.6–1.78)	0.875
No activity	1.75 (0.73–4.16)	0.206	1.36 (0.78–2.38)	0.273
Diabetes				
No	1.00		1.00	
Yes	1.47 (0.63–3.39)	0.371	0.70 (0.22–2.21)	0.537
Asthma				
No	1.00		1.00	
Yes	0.84 (0.40–1.78)	0.653	1.12 (0.63–1.98)	0.701
Arthritis				
No	1.00		1.00	
Yes	1.80 (0.81–3.99)	0.146	1.03 (0.64–1.63)	0.915
Cardiovascular disease				
No	1.00		1.00	
Yes	1.61 (0.76–3.41)	0.218	1.23 (0.57–2.65)	0.604
Anxiety				
No	1.00		1.00	
Yes	0.35 (0.05–3.41)	0.307	1.67 (0.69–4.06)	0.255
Depression				
No	1.00		1.00	
Yes	0.79 (0.29–2.10)	0.630	0.98 (0.43–2.27)	0.970
Stress				
No	1.00		1.00	
Yes	2.19 (0.94–5.72)	0.109	1.24 (0.42–3.67)	0.702
General health				
Excellent/very good/good	1.00		1.00	
Fair/poor	0.82 (0.34–1.94)	0.645	1.24 (0.73–2.11)	0.418
Systolic blood pressure	0.96 (0.94–0.99)	0.011	0.99 (0.97–1.00)	0.133
Diastolic blood pressure	1.13 (1.07–1.20)	<0.001	1.02 (0.98–1.05)	0.357
CRP	1.03 (1.00–1.06)	0.023	1.00 (0.94–1.06)	0.971
HbA1c	0.54 (0.33–0.89)	0.016	1.11 (0.67–1.84)	0.681
HDL	1.03 (0.36–2.92)	0.963	1.40 (0.81–2.40)	0.226
LDL	1.09 (0.81–1.46)	0.576	1.15 (0.90–1.47)	0.257
Glucose	1.40 (1.03–1.89)	0.031	0.90 (0.64–1.27)	0.551
Hb	0.98 (0.95–1.00)	0.087	1.00 (0.97–1.02)	0.715
Vitamin D	1.00 (0.99–1.01)	0.516	0.99 (0.99–1.00)	0.138
Albumin	0.90 (0.82–1.00)	0.040	0.97 (0.89–1.05)	0.419
IL-6	0.75 (0.57–1.00)	0.051	0.97 (0.83–1.14)	0.707
TNFα	1.12 (1.06–1.20)	<0.001	1.03 (0.98–1.07)	0.242
MPO	1.00 (1.00–1.00)	0.895	1.00 (1.00–1.00)	0.657
eSel	0.99 (0.97–1.00)	0.140	1.02 (1.00–1.03)	0.115

## Discussion

Reviewing our research questions, we examined whether informal family carers showed different biomedical profiles in terms of blood and other measured variables than non-carers. Overall our carers aged 40 years and over had only slightly elevated levels of the inflammatory biomarkers TNFα, hs-CRP, and HbA1c but they showed lower Vitamin D and Hb levels. The second research question investigated if there was an association between the caregiving role, risk factors and chronic conditions amongst informal carers. Our findings indicate that when carers were compared with non-carers, they were more likely to have higher BMI and WHRs, report less than optimal physical activity and describe their health status as fair/ poor. In terms of chronic conditions carers were more likely to report diabetes, arthritis, anxiety and depression than non-carers. However stress-related conditions were not evident amongst carers in our study and they reported significantly lower or no alcohol consumption risk (p = 0.041). They were also less likely to be current smokers.

### Vitamin D and other blood measured variables

Comparing the large number of haematological and biochemical variables of carers with non-carers in the NWAHS, yielded a few differences in blood pictures, for instance, levels of Vitamin D, Hb, HbA1c, TNFα and hs-CRP. Most of these results were within acceptable ranges, but of the five blood measured variables of interest, 25(OH)D (Vitamin D) was the most notable result showing that carers had lower median levels when compared with non-carers. Despite a large body of research on Vitamin D in the biomedical literature, of the studies collated, no comparable clinical research and population surveys could be identified reporting any association of Vitamin D with carer health outcomes in the context of informal caregiving. One previous project involving Stage 3 participants of the NWAHS, although not specific to carers, does provide an insight into Vitamin D and associated characteristics of that population [45]. Obesity (indicating higher BMI), physical activity, gender and seasonality all appeared to have a strong association with Vitamin D levels. For instance participants had lower Vitamin D levels even with seasonal variations during summer / spring months [45]. In our study based on participants from the same NWAHS population, carers had lower levels of Vitamin D in comparison to non-carers. This finding is important as Vitamin D can prevent conditions such as osteoporosis, particularly in post-menopausal women who represent up to half of all the female carers. Although osteoporosis was not significant amongst carers in our study, if carers were to develop osteoporosis related to low Vitamin D and nutritional deficiencies, they could be more at risk of injuries from frequently moving and lifting the persons being cared for [3]. Haemoglobin was found to be statistically different between the NWAHS carers and non-carers. Albumin, another blood measured variable, was lower amongst male carers who were more at risk of lower levels than non-carers (RR = 0.90, CI 95% 0.82–1.00; p = 0.040) however these levels were not of clinical significance. Again, of the studies reviewed in the literature, none specifically highlighted haemoglobin or albumin in carer populations. Several studies reported measuring haematological and serum chemistry in carers as part of larger projects but, found few notable differences between the full blood counts with non-carers, other than variables specific to their own studies [46,47].

### Inflammatory biomarkers: TNFα and hs-CRP

In our study there were only slight statistically significant differences in blood measured inflammatory biomarkers amongst NWAHS carers aged 40 years and over. Namely, the immune regulatory cytokines hs-CRP and TNFα. These are acute phase markers of inflammation, especially hs-CRP which is used as a non-specific but very sensitive biomarker for detecting systemic inflammatory conditions, tissue damage and infection, as well as early onset cardiovascular disease [48]. Although inflammatory biomarkers are not as frequently assessed in biomedical studies of caregivers, previous studies have identified male carers as more vulnerable to physiological and pathological changes as predicted by the presence of hs-CRP [25,49,50]. Our NWAHS male carers had minimally raised plasma levels of inflammatory biomarkers TNFα and to a lesser extent, hs-CRP when compared to non-carer male but the cytokine IL-6 levels were much lower in male carers when compared with male non-carers (RR 0.75, 95% CI 0.75–1.00; p = 0.051) (See Table 4). Von Kanel et al (2012) had observed that being a carer did not necessarily show increased hs-CRP levels, but rather hs-CRP increased over time as the caregiving burden continued. The longer duration of caregiving with elevated biomarkers (TNFα and hs-CRP) suggested a pro-inflammatory state [25]. As we did not have equivalent carer details in our own study we were unable to examine biomarkers in terms of the duration of caring to make a comparison.

### HbA1c, Type II diabetes and related chronic conditions

Blood measured HbA1c levels were inconsistent in our study. Glycated Haemoglobin (HbA1c) is a measure that provides information on long-term glucose control. HbA1c, is a recognised biomarker used to establish the prevalence or presence of Type 2 diabetes. Our findings showed significant but minimal differences in HbA1c blood levels in our sample of carers. However self-report data suggested carers were at greater risk of Type 2 diabetes than non-carers. This was a similar finding to a previous state-wide population survey also using self-report data from South Australian carers which we conducted between 2010–2015 [51]. Few published Australian population surveys that included carers have featured specific chronic conditions such as diabetes [20], while self-report health surveys conducted in Brazil, the USA and other countries have reported Type 2 diabetes in informal carers. In the biomedical literature there has been limited attention given to investigating diabetes-related characteristics in carers, and with the exception of one study [52], the emphasis has usually been on Type 2 diabetes in the person being cared for, rather than the carer.

### Risk factors: Physical activity and BMI

Carers in our study were more likely to report insufficient activity or “no activity” than non-carers, but how participants interpreted the questions about their own physical activity may have been a factor in their responses. For example, carers might be physically active but have a different type and level of activity associated with demanding caregiving duties. Older carers in particular and those with their own disabilities may not be able to participate in recreational exercise or sport. This was partly investigated in a population based study which included community dwelling informal carers aged 40 years and over (n = 1380) from the German Ageing Survey [53]. They found decreased sporting activities and higher BMI amongst carers could lead to adverse health outcomes for carers. It was also concluded that time spent caregiving performing regular personal care activities and nursing care services for persons in a poor state of health could be associated with stress and depression, which can in turn be linked with higher BMI [53]. These results are in keeping with trends from our own research confirming higher BMI in carers [54].

### Stress and anxiety

In the caregiving literature, parent carers, dementia and mental health carers have reported lower perceived health status [55,56]. NWAHS carers were also more likely to state their health was fair/poor. From a large British study of over 8000 middle aged men and women, carers rated their physical or mental health as fair/poor however it was further suggested that the rating of *poor* health could be ‘proxy markers of perceived stress’ [57]. Our findings from NWAHS carers overall did not specifically indicate carer-related stress which was unexpected, but anxiety and depression were two other aspects of psychological morbidity identified amongst our carers. Sherwood et al had found a significant association between anxiety in male carers of spouses with cancer and anxiety was seen as a risk factor for higher levels of inflammation in male carers [58].

Female carers when compared with female non-carers in our study had fewer significant risk factors or chronic conditions, but male carers presented quite a mixed biomedical profile when compared with non-carer males. The female carers in our study tended towards more metabolic and anthropometric manifestations that suggested a stronger association with BMI and adiposity. Kang et al however had found that while there was an association of metabolic dysfunction with family caregiving, no gender differences emerged from their large national study [59].

### Strengths and limitations

The main strengths of this study are that both biomedical and self-report data were obtained from a large sample of metropolitan residents. It included a substantial number of blood tested and measured variables which were collected at clinics and during interviews. Clinically accessed information was a central part of this study which included a wide range of observed and measured variables for major risk factors, seven chronic conditions, inflammatory and other biomarkers. Carers in this study were more heterogeneous than recruited participants as they did not represent any one particular group of people living with specific disabilities or medical conditions. This type of large population study is usually cost prohibitive and requires the collaboration of a consortium of academic and government groups. It therefore offers a more comprehensive review of carer health characteristics than is usually possible.

There were limitations however in relation to the assessment of carer participants identified and grouped as a subset of this cohort study so they could be compared with the non-carers within the same population. The definition of *informal carer* chosen to identify carers was the standard used within Australia, however other carer specific questions were not included to further classify the type of caregiving. Within this study therefore we do not have details of the cared for person’s age, diagnosis, health, disability or disease status, and their level of dependency, all of which have been reported as impacting on the role as informal carer. Nor was information collected on duration of caring—for example how long spent caring; how many hours per day or per week they were providing care and the level of intensity of their caring role. Further we do not have information on whether the participant was the main carer; if they were co-resident with the person being cared for; what other caregiving demands were put on the carers and which carers were combining personal caregiving with paid employment. Another weakness of this study is that we do not know which conditions reported by the carers, were pre-existing and therefore whether the risk factors and chronic conditions could be actual health outcomes of the caregiving experience. Also we do not know the severity of carers’ illnesses and if they had multiple health problems as not all potential chronic conditions were included in this research. The lack of biomedical data on those aged less than 40 years is also a weakness of the study.

### Conclusions and recommendations

Our study has demonstrated that in terms of blood and other clinic measured variables the NWAHS carers did show some differences in their biomedical health profiles when compared with non-carers. In contrast to other published studies our findings suggest carers may be at risk of lower Vitamin D and Hb levels thus revealing a possible gap in current knowledge of carer morbidity. It is acknowledged that the differences in other blood measured variables were minimal when compared with non-carers, but the significance of lower Hb, raised TNFα as well as hs-CRP in male carers highlights the need for ongoing assessments of their biomedical health status [60].

From a population perspective, urban carer participants’ results indicated that there are carers providing care in less than optimum health, reporting chronic conditions of diabetes, arthritis, anxiety and to a lesser extent, depression. These cross-sectional analysis results provide only weak associations between the caregiving role, risk factors and chronic conditions. In contrast to previous studies, stress was not a significant finding. Higher BMI amongst carers generally, and particularly in female carers, combined with other risk factors such as insufficient physical activity, warrants closer scrutiny. Carers may have less opportunities to undertake physical activity, have less time outdoors and consequently less exposure to Vitamin D and less opportunity to maintain a healthy weight. Public health strategies targeting carers and addressing these factors may be worthy of consideration. Our research therefore recommends closer monitoring of carer health and morbidity trends across populations over time with special attention to the choice of health variables requiring ongoing measurement and assessment. This would contribute to the development of more balanced health policies and clinical guidelines for chronic diseases that are carer specific and age sensitive. Policymakers and health professionals therefore need to take into account the differences in carer health status, risk factors and morbidities for male and female carers.

## Abbreviations

BMI: Body Mass Index
CATI: Computer Assisted Telephone Interview
CI: Confidence interval
CRP: C-Reactive Protein
CVD: Cardiovascular Disease
e-Sel: e-Selectin
FEV1: Forced Expiratory Volume in one second
Hb: Haemoglobin
HbA1c: Glycosylated haemoglobin
HDL: High Density Lipoprotein
hs-CRP: high sensitivity C-Reactive Protein
Il-6: Interleukin-6
LDL: Low Density Lipoprotein
MPO: Myeloperoxidase
NWAHS: North West Adelaide Health Study
RR: Relative risk
SF1: Short Form 1 (Question 1)
SF36: Short Form 36 (Questionnaire)
SPSS: Statistical Package for Social Sciences
TNFα: Tumor Necrosis Factor alpha
WHR: Waist Hip Radio
